# Predictive Analysis of Hospital Stay after Caesarean Section: A Single-Center Study

**DOI:** 10.3390/bioengineering10040440

**Published:** 2023-04-01

**Authors:** Alfonso Maria Ponsiglione, Teresa Angela Trunfio, Francesco Amato, Giovanni Improta

**Affiliations:** 1 Department of Electrical Engineering and Information Technology, University of Naples Federico II, 80125 Naples, Italy; 2 Department of Advanced Biomedical Sciences, University of Naples Federico II, 80131 Naples, Italy; 3 Department of Public Health, University of Naples Federico II, 80131 Naples, Italy; 4 Interdepartmental Center for Research in Healthcare Management and Innovation in Healthcare (CIRMIS), University of Naples Federico II, 80131 Naples, Italy

**Keywords:** machine learning, caesarean section, length of stay

## Abstract

Caesarean section (CS) rate has seen a significant increase in recent years, especially in industrialized countries. There are, in fact, several causes that justify a CS; however, evidence is emerging that non-obstetric factors may contribute to the decision. In reality, CS is not a risk-free procedure. The intra-operative, post-pregnancy risks and risks for children are just a few examples. From a cost point of view, it must be considered that CS requires longer recovery times, and women often stay hospitalized for several days. This study analyzed data from 12,360 women who underwent CS at the “San Giovanni di Dio e Ruggi D’Aragona” University Hospital between 2010 and 2020 by multiple regression algorithms, including multiple linear regression (MLR), Random Forest, Gradient Boosted Tree, XGBoost, and linear regression, classification algorithms and neural network in order to study the variation of the dependent variable (total LOS) as a function of a group of independent variables. We identify the MLR model as the most suitable because it achieves an *R*-value of 0.845, but the neural network had the best performance (*R* = 0.944 for the training set). Among the independent variables, Pre-operative LOS, Cardiovascular disease, Respiratory disorders, Hypertension, Diabetes, Haemorrhage, Multiple births, Obesity, Pre-eclampsia, Complicating previous delivery, Urinary and gynaecological disorders, and Complication during surgery were the variables that significantly influence the LOS. Among the classification algorithms, the best is Random Forest, with an accuracy as high as 77%. The simple regression model allowed us to highlight the comorbidities that most influence the total LOS and to show the parameters on which the hospital management must focus for better resource management and cost reduction.

## 1. Introduction

In the last few years, one of the main relevant procedures is the Caesarean section (CS), which is the one used to deliver a foetus. It involves making an initial incision in the abdomen (laparotomy) and a subsequent one in the uterus (hysterectomy) [1]. Despite being a surgical procedure, the CS rate has increased in recent years, especially in industrialized countries [2]. For example, in the United States, it is the most commonly performed surgery, involving about one million women, with a rate of 31.9% [3]. In turn, there was a volume of hospitalizations for CS in Italy equal to 131,390 in 2020, a slight decrease compared to the previous year. [4] Regarding primary CS, the frequency is almost stable, from a median value of 23.6% in 2015 to 22.0% in 2020 [4]. Campania has the highest rate of CS among Italian regions, with a percentage of 58.4%. Additionally, in private hospitals that perform fewer than 500 deliveries annually, this rate was found to be as high as 84.4% [5].

Although efforts are underway to reduce the CS rate, experts do not predict a significant decline for at least a decade or two [6]. In fact, there are several causes that justify a CS. Previous perineal trauma, cardiac or pulmonary disease, placental abruption, and umbilical cord prolapse are just a few examples of causes that justify its use [7,8]. However, evidence is emerging that non-obstetric factors may contribute to the decision [9,10]. Scientific advances, as well as legislative and social changes, have affected the attitude towards CS. The increase in CS rates is attributed to various psychosocial factors, such as maternal anxiety about a delivery, a preference for CS without medical indication, a rising number of older first-time mothers, and the perception that the procedure is free of risks [11]. In reality, CS is not a risk-free procedure. Intraoperative risks, such as infections, organ injury or transfusion, thromboembolic complications, or risks for a subsequent pregnancy, such as placenta previa or infertility, are possible complications. Children are also at risk of bronchial asthma, type 1 diabetes mellitus, or allergic rhinitis [12,13,14,15]. In 2010, the World Health Organization (WHO) stated that CS compared to vaginal delivery (VD), is associated with a higher risk for the mother and baby and, therefore, should be performed only in cases of extreme necessity [16].

It should be considered that CS requires longer recovery times, and women often remain hospitalized for several days [17]. Hospital length of stay (LOS) is often used as a quality indicator for healthcare processes. For example, introducing a faster clinical care pathway jointly reduces the length of hospital stay (LOS) and hospitality expenses, as shown in [18]. Ferraro et al. [19], on the other hand, utilize the Lean Six Sigma approach to analyze the impact of healthcare-associated infections using LOS as a control variable. Strategies need to be implemented to objectively study healthcare processes [20,21,22] or support resource management to contain costs in an increasingly business-like healthcare system [23,24,25,26]. In procedures such as childbirth, expenditure items are mainly associated with the hospitalization of the mother and child, the use of neonatal intensive care, and the type of delivery [27]. Early discharge after childbirth has become an increasingly common practice. Early discharge is defined as when the LOS is less than 2 days for natural delivery and 4 days after CS [28,29]. Being able to standardize LOS can help not only to keep expense items constant but also to support scheduling and planning activities, which are particularly important for elective surgery. For these reasons, it becomes strategic to know the variables that influence LOS.

In this work, an extensive predictive analysis is conducted to model the LOS of women who underwent CS at the “San Giovanni di Dio e Ruggi D’Aragona” University Hospital. Different machine learning algorithms have been applied to address both regression and classification tasks, and results were systematically analyzed and compared in terms of performance metrics in order to find the most suitable approach to model the LOS, which represents a primary and among the most relevant indicator of the service quality in healthcare organizations. The study mainly contributes to the investigation and identification of the most promising clinical and organizational decision-support strategies based on the use of artificial intelligence tools in clinically relevant settings. Indeed, the ability to select the most valuable and powerful algorithms to predict LOS in advance, with an acceptable and tolerable margin of error, might be a useful tool for improving the management of costs and complexity in hospitals as well as for evaluating proper resource usage and allocation. At the same time, however, the strategies should be easy to be implemented in healthcare structures and should rely on readily available data such as those collected in electronic health records or administrative databases. In this regard, this work proposes and investigates data mining strategies based on using standardized and computationally efficient machine learning methods that can be fed with data and information available in the most widespread healthcare information systems. This study extends a previously published study [30] in which MLR was used to build a preliminary model based on a limited number of years (2019–2020) and on a limited number of variables with an R^2^ value of 0.925. In particular, a more detailed analysis of comorbidities will allow a better classification and understanding of the factors that most influence total LOS.

### Related Works

Innovation in the field of data analysis techniques, which achieved high performances in different domains [31,32,33,34,35,36], had a significant impact on healthcare.

These tools, despite the problems related to security due to the particular field of application [37], starting with applications such as the analysis of biomedical data [38,39,40] or support for the diagnosis and treatment of diseases [41], are also spreading in hospital resource management and more generally in healthcare management. Ponsiglione et al. [42], for example, use a Finite-State Machine to investigate the phenomenon of drop-out from Medical Examinations. Huyen et al. [43], on the other hand, use both an autoregressive integrated moving average (ARIMA) model and a geographic information system (GIS) to analyze hospital-cost payments of patients treated as a function of geographic area from a teaching hospital in Vietnam.

Optimizing costs and health care also involves optimizing processes and, thus, patient flow within the hospital [44].

In the context of CS, these techniques have been successfully implemented in different aspects. Chai et al. [45] use the DMAIC cycle and Lean Six Sigma methodology to identify causes and thus reduce the rate of CSs, while Verhoeven et al. [46] use logistic regression-based models to discriminate whether or not to perform CS from induced labor. The review conducted by Deng et al. [47] shows us that logistic regression models are the most widely used models in the literature to study and predict VD after CS, using predictors such as body mass index, previous vaginal delivery, and maternal age. As performed by Ehrenberg et al. [48], model predictors could also be used to identify major risk factors to analyze the impact of Diabetes or Obesity on the risk of performing CS.

Returning to the topic of our work, the study of LOS, several works have been conducted in Italy. Scala et al. [49], for example, use multiple linear regression and classification algorithms to predict the LOS of patients who accessed the hospital for a lower limb fracture, while Olivato et al. [50] use machine learning algorithms to assess the LOS of hospitalized patients with COVID-19.

As for CS, except for the one conducted by our research team [30,51] on a small number of variables and years of observation, we are not aware of any other work to date.

## 2. Materials and Methods

This study analyzed data from 12,360 women who underwent CS at the “San Giovanni di Dio e Ruggi D’Aragona” University Hospital between 2010 and 2020, extracted from the QuaniSDO information system, which is in use for the computerization of hospital discharge forms. In particular, the following variables were extracted:

- Age;
- Date of admission, discharge, and CS procedure;
- Primary and secondary diagnoses;
- Diagnosis-related group (DRG);

Through a study of the DRGs, it was possible not only to discriminate the CS from the VD but also to identify the presence or absence of complications during the procedure. From the study of principal and secondary diagnoses, major comorbidities and conditions were extracted, and the dataset was divided into multiple subgroups of patients with similar conditions. From date extraction, total LOS (the dependent variable) and the Pre-operative LOS were calculated. After this preliminary elaboration, the independent variables of the model were as follows:

- Age;
- Pre-operative LOS,
- Thyroid disorder (yes/no);
- Cardiovascular disease (yes/no);
- Abnormal foetus (yes/no);
- Respiratory disease (yes/no);
- Hypertension (yes/no);
- Diabetes (yes/no);
- Haemorrhage (yes/no);
- Brain and retinal disorders (yes/no);
- Multiple births (yes/no);
- Obesity (yes/no);
- Amniotic fluid disorders (yes/no);
- Stillborn (yes/no);
- Pre-eclampsia (yes/no);
- Tumour (yes/no);
- Complicating previous delivery (yes/no);
- Urinary and gynaecological disorders (yes/no);
- Complication during surgery (yes/no).

**Figure 1:**
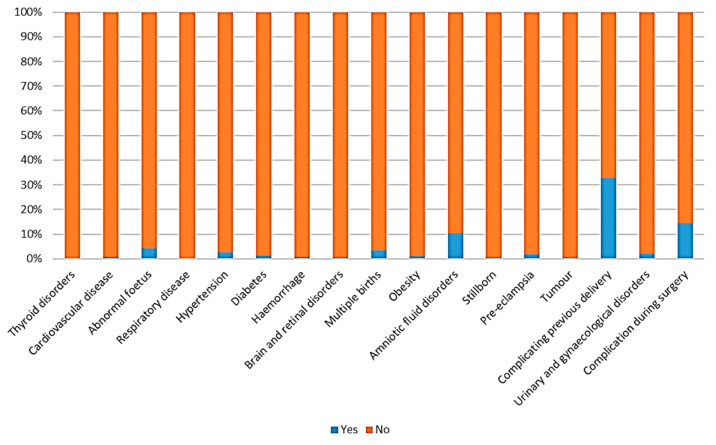
Dichotomous characteristics of the dataset.

Figure 1 shows the characterization of the categorical variables in the dataset.

### 2.1. Regression Models

IBM SPSS (Statistical Package for Social Science) ver. 20 and KNIME Analytics Platform ver. 4.3.2 were used to implement Regression models. A multiple linear regression (MLR) model was built with IBM SPSS. Before implementing and evaluating the performance of the model, it is necessary to verify six preliminary hypotheses, i.e., the linearity relationship between dependent variable (total LOS) and independent variables (Age, Pre-operative LOS, Thyroid disorder, Cardiovascular disease, Abnormal foetus, Respiratory disease, Hypertension, Diabetes, Haemorrhage, Brain and retinal disorders, Multiple births, Obesity, Amniotic fluid disorders, Stillborn, Pre-eclampsia, Tumour, Complicating previous delivery, Urinary and gynaecological disorders, Complication during surgery), absence of multicollinearity and outliers and some properties of the residues. If these hypotheses are verified, it is possible to proceed with the use of a linear model for problem characterization. KNIME Analytics Platform is instead used to test additional regressive algorithms. Random Forest (RF) is an algorithm for supervised learning that leverages the combination of multiple learning algorithms to enhance its performance. Although the resulting model is both powerful and precise, there is a considerable likelihood of overfitting. Gradient Boosted Tree (GBT) is a statistical learning algorithm that operates without a fixed set of parameters and can be employed for both regression and classification problems. Similar to RF, it creates a decision model that consists of a sequence of basic forecasting models, usually decision trees. These models are incrementally integrated into each step to improve the output of the prior Weak Learner. The XGBoost algorithm is a gradient-boosting technique that can be applied to predictive regression modeling. As with the previously mentioned algorithms, it involves the iterative incorporation of decision trees to enhance the accuracy of the previous model. Furthermore, the XGBoost algorithm utilizes any differentiable loss function and a gradient descent optimization algorithm for fitting models. Consequently, the method is termed “gradient boosting” because it aims to minimize the loss gradient during model fitting. Logistic Regression (LR) is a model building a linear relationship between the input and output variables. There are various approaches to training the linear regression equation using data, with the most prevalent method being ordinary least squares. This approach entails estimating the coefficients’ value from the data available during the learning process. For each artificial intelligence model, a partition of 80% was employed to create the training dataset, while the remaining 20% was allocated for the test set.

### 2.2. Classification Algorithms and Neural Network

Another way to investigate total LOS is through the implementation of classification algorithms. To do so, it is necessary to define the dependent variable not continuously but through homogeneous classes. In accordance with the literature [28,29], the total LOS was divided into three classes as follows:

- Group 0: 0–4 days;
- Group 1: 5–6 days;
- Group 2: LOS > 6 days.

Google Colaboratory (Colab) Cloud Platform [52] was chosen for the implementation. The selected classification algorithms are Decision Tree (DT), Random Forest (RF), Support Vector Machine (SVM), Naïve Bayes (NB), and Multilayer Perceptron (MLP).

DT puts simple decision trees at the basis of the classification process, which is then improved by more complex algorithms such as RF, discussed extensively in the previous section. In contrast, a different approach is used by SVM, NB, and MLP. SVM bases the classification process on finding the best hyperplane for data separation, while NB is a statistical classifier based on Bayes’ theorem, albeit assuming the simplifying assumption of class conditional independence as the basis. Finally, MLP is a feed-forward neural network supplement composed of neurons called perceptrons that receive weighted features as input and, through activation functions, produce the output. Learning, in this case, consists of adjusting these weights with the goal of minimizing a specific parameter, which in this case is the mean square error. In addition to these algorithms, Voting Classifier (VC) was used to combine performance and obtain a better classifier. To do this, a majority policy is implemented; that is, the predicted value from at least 3 classifiers will be associated.

For the implementation of the algorithms, it was decided to make a partition with 80% of the data for the training set and 20% for the test set. However, this partitioning is not static. Using CrossValidator belonging to the scikit-learn library used to design artificial algorithms, which have been above defined, the dataset was partitioned into *N* = 10 pairs of separate datasets (training, test) to analyze the effectiveness of models according to a predefined set of parameters. The performance of the models will be identified as the average of the values obtained on the single partition. In addition to this, GridSearchCV tool was used for optimization of hyperparameters of the selected algorithms. This makes it possible to adjust the parameters to the particular data set. Table 1 shows the parameters that were arbitrarily selected for the above-defined artificial intelligence models.

**Figure 2:**
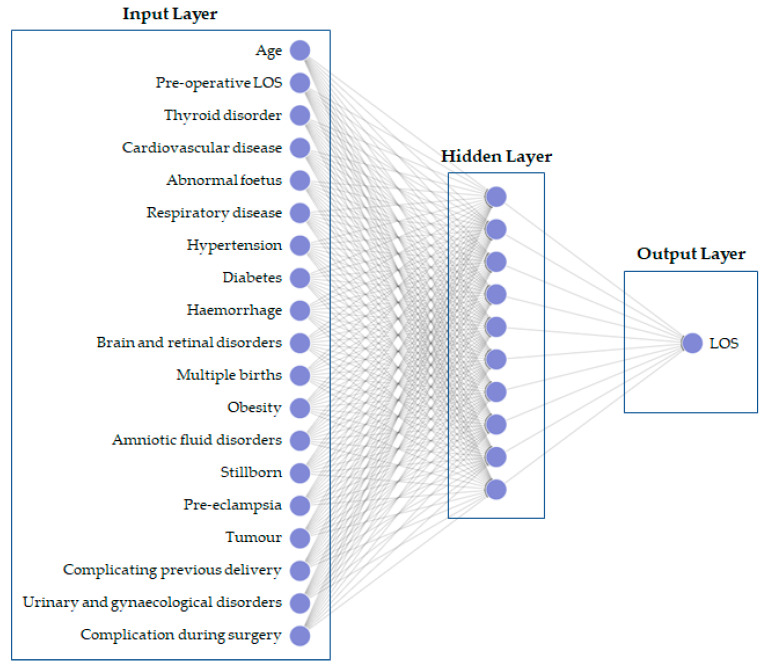
Artificial neural network architecture.

Lastly, MatLab version R2020a was used to implement the neural network (NN). The network implemented was a 2-layer feed-forward network with two different transfer functions. In the hidden layer, there was a sigmoid transfer function, while in the output layer a linear transfer function. In addition, in the hidden level, the number of hidden neurons was ten. Figure 2 shows the network architecture.

The Levenberg–Marquardt algorithm was used for the training. In fact, this algorithm is recommended for most problems, requiring more memory but less time. The training stops automatically when there is an increase in the mean square error of the validation samples. Training continues until the validation error increases consecutively for six iterations. The dataset has been split into three sub-sets: training (70%), validation (15%), and test (15%).

## 3. Results

**Figure 3:**
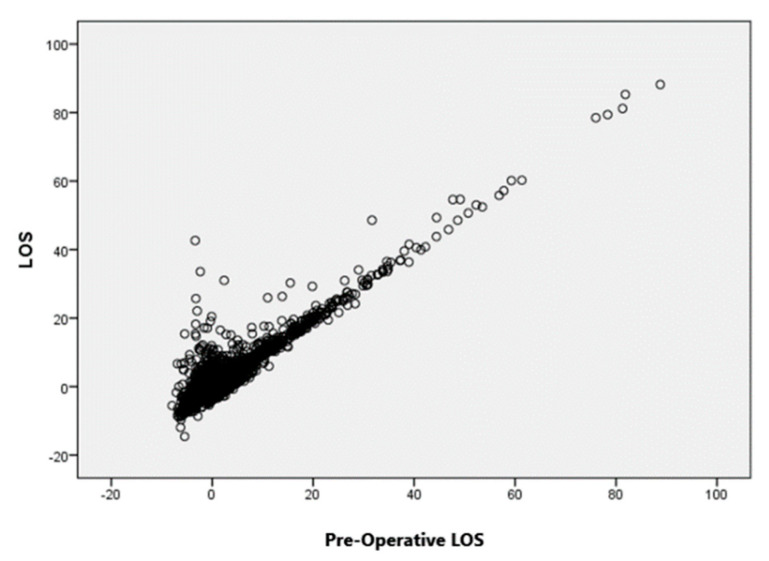
Partial regression plot of the multiple linear regression model.

After preliminary processing of the dataset to obtain the set of independent variables, The hypotheses for employing the multiple linear regression (MLR) model were validated by examining the linear relationship between the dependent variable and the independent variables using appropriate scatter plots, such as the one depicted in Figure 3.

In this case, the linear relationship is clearly evident, also in agreement with the definition of total LOS. This type of plot did not allow evaluation of the effect generated by the simultaneous interaction of multiple input variables.

As for the residues, their independence was verified through the Durbin–Watson test. The result, 1.853, is contained within the acceptance range of (1.5; 2.5) required by the test. As for the variance, on the other hand, its constant trend is verified through the creation of a scatter plot showing the “standardized expected value regression” on the *x*-axis and the “standardized residual regression” on the *y*-axis.

**Figure 4:**
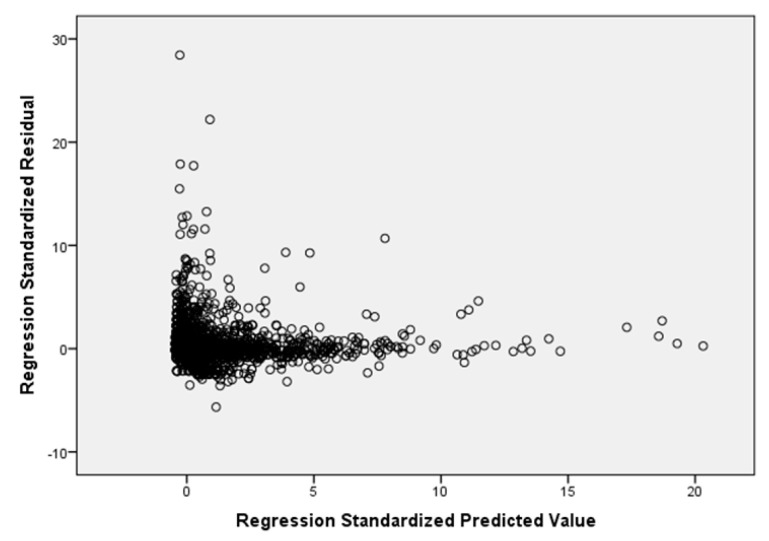
“Standardized expected value regression” vs. “Standardized residual regression” of the multiple linear regression model.

**Figure 5:**
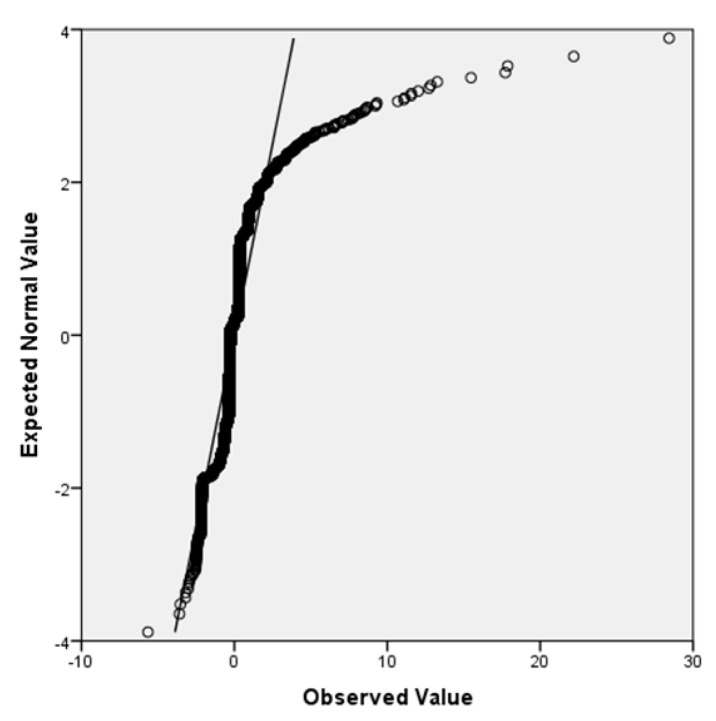
Q-Q plot of the standardized residuals of the multiple linear regression model.

In Figure 4, the data are randomly distributed around zero. It was shown that the assumption of homoscedasticity is not violated. Finally, the normality of the distribution was always verified graphically using the Quartile–Quartile plot shown in Figure 5.

The majority of the points were situated near the solid line, representing the ideal trend, with only a few outliers that did not affect the model’s goodness of fit. To ensure the absence of multicollinearity, two parameters—Tolerance and Variance Inflation Factor (VIF)—were utilized, both of which are dependent on the correlation between the *i*-th independent variable and the others. Cook’s distance was also calculated for each observation to verify that there were no outliers that could impact the estimation of the model parameters.

In Table 2, multicollinearity was confirmed to be absent as the VIF values were consistently under 10 and the Tolerance values were consistently above 0.2. Cook’s distance, on the other hand, was less than 1 for each of the 12,360 observations, guaranteeing the absence of outliers. After this phase, the MLR model was implemented, and its performances are shown in Table 3.

Even on a dataset consisting of several observations and different independent variables, the excellent performance of the MLR model is demonstrated by an R^2^ parameter above the limit value of 0.5. Table 4 shows the calculated coefficients and the result of the *t*-test. The significance level chosen is 0.05, and the purpose is to highlight which variables significantly influence the output.

From Table 4, it is highlighted that the variables that most influence total LOS were Pre-operative LOS Cardiovascular disease, Respiratory disorders, Hypertension, Diabetes, Haemorrhage, Multiple births, Obesity, Pre-eclampsia, Complicating previous delivery, Urinary and gynaecological disorders and Complication during surgery. Among these, the highest coefficient is associated with Pre-operative LOS.

Table 5 shows the effectiveness performances of other regression models in terms of R^2^ and Root Mean Squared Error.

GBT achieved the highest performance among the tested algorithms with an R^2^ value of 0.844, followed by LR with 0.839, XGBoost with 0.838, and finally, RF with 0.705.

After completing the study with the regression models, we moved on to the implementation of the classification models. Table 6 shows the results in terms of accuracy and the optimized parameters for the particular dataset used.

In terms of accuracy, the best algorithm is RF, followed by DT. Ultimately, decision trees proved to be the best in predicting total LOS. Even VC could not improve performance by establishing in definitive what RF is the best algorithm. Table 7 shows the additional parameters for the best algorithm.

**Figure 6:**
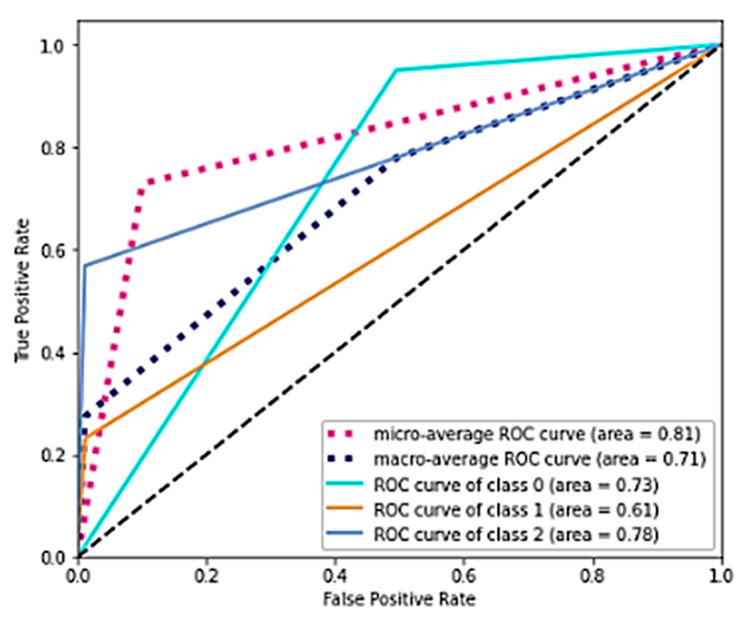
ROC curves. The discontinuous black line represents the "no benefit" line, i.e., a causal classifier with area = 0.5.

The results by individual class showed that the worst results were obtained in the intermediate class. In contrast, excellent results were obtained for class 0 and class 2. This finding is not insignificant as class 0 is the most representative of the sample (*N* = 7834), while class 2 is the most critical for healthcare management as it encloses women with prolonged hospitalization. The same result is shown graphically with the ROC curves in Figure 6.

**Figure 7:**
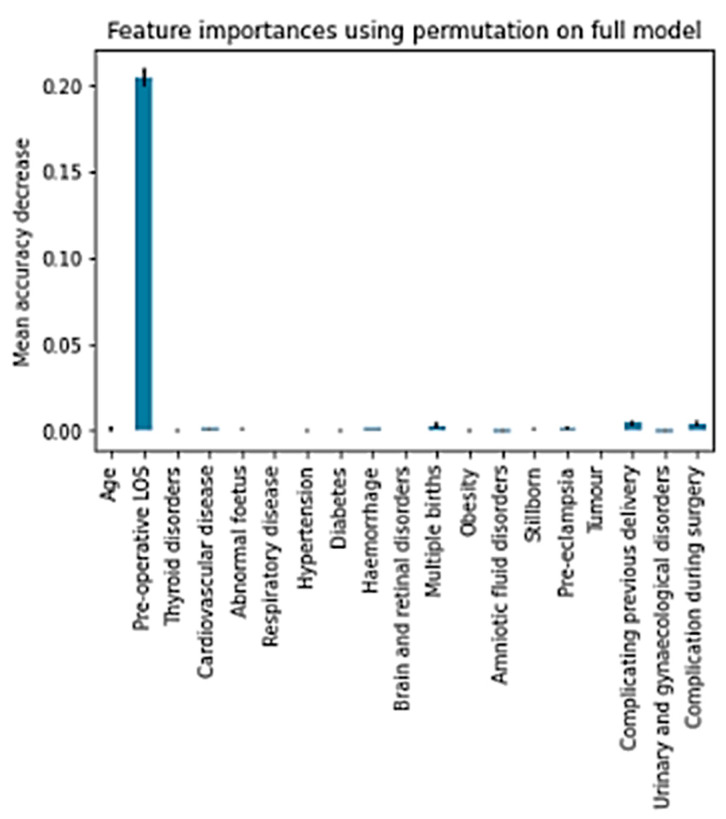
Permutation Feature Importance.

As anticipated, the minor area with respect to the black characteristic of “no benefit” was precisely that associated with class 1. However, the micro and macro average values showed an area above 0.7. Feature importance permutation was used to evaluate the effect of the independent variables on classification. This procedure consists of evaluating the performance of the algorithm by going to corrupt any of the independent variables one by one. Figure 7 shows how much the accuracy is lowered due to the corruption of a specific independent variable.

The graph shows that the only significant effect is related to Pre-operative LOS, which is part of the overall LOS by definition. Other effects, albeit insignificant, are associated with Multiple Births, Complications from previous delivery, and Complications during surgery. Lastly, the NN Fitting was implemented. Table 8 shows the results obtained.

**Figure 8:**
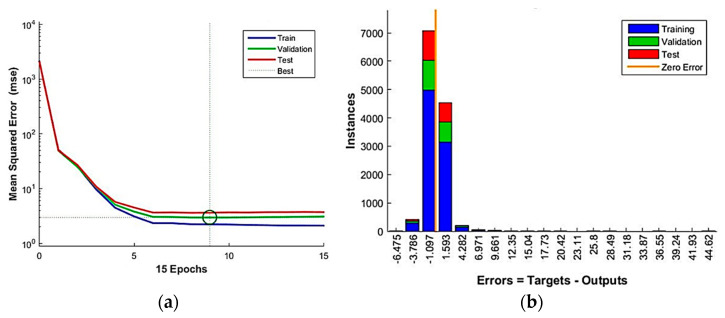
(**a**) Training process and (**b**) error histogram of the employed artificial neural network.

As regards the MSE, Figure 8 illustrates the training process and the error of the proposed artificial neural network by displaying the trend curves of the MSE as a function of the epochs (Figure 8a) and the histogram of error distribution (Figure 8b) for both training, validation, and test subsets.

Figure 8a shows how the training of the proposed artificial neural network with the best performance, in terms of MSE, obtained after 9 epochs, where each of the three curves (for train, test, and validation subsets) reach the best value of the MSE, equal to 2.96, following a similar trend. Figure 8b shows the error histogram of the implemented model, where the highest bars are narrowly distributed around the zero-error (solid line) with a moderately long right tail on a limited number of instances.

**Figure 9:**
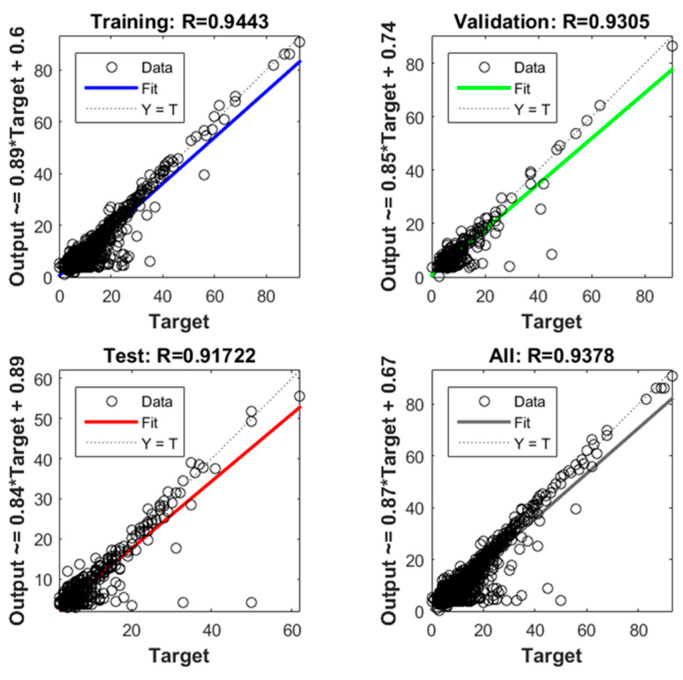
Regression plots for the proposed artificial neural network.

Figure 9 shows the regression plots from the implemented regression model based on the proposed artificial neural network for both training, validation, and test subsets.

The scatter plots in Figure 9 display the predicted LOS values on the *y*-axis against the actual LOS values on the *x*-axis for both the training, test, validation subsets as well as for the and overall dataset. The linear fitting curve (solid line) is also reported for each plot along with the identity line (dashed line), representing the optimal agreement between real and predicted data. As can be observed, the obtained linear fitting curves are close to the identity line, with correlation coefficients (R) equal to 0.94, 0.93, and 0.92 for training, validation, and test data, respectively, and with the overall R of the model equal to 0.94, thereby indicating the quality of the artificial neural network and its promising predictive power.

## 4. Discussion

In this paper, data on CSs at the “San Giovanni di Dio e Ruggi d’Aragona” University Hospital were analyzed. In particular, the information of 12,360 women who had a CS in the years 2010–2020 was extracted from the QuaniSDO information system. Starting from a restricted set of variables, such as Age, DRG, Date of admission, Date of CS, and Date of discharge, the dependent variable (total LOS) and independent variables (Age, Pre-operative LOS, Thyroid disorder, Cardiovascular disease, Abnormal foetus, Respiratory disease, Hypertension, Diabetes, Haemorrhage, Brain and retinal disorders, Multiple births, Obesity, Amniotic fluid disorders, Stillborn, Pre-eclampsia, Tumour, Complicating previous delivery, Urinary and gynaecological disorders, Complication during surgery) were obtained. From these, an MLR model was constructed to provide the hospital with a tool to first determine the LOS based on the variation in one or more independent variables. The resulting model produced an R^2^ value of 0.876. The good performances are in line with the results already obtained on a sample based on 1817 women undergoing CS in the years 2019–2020. In this case, in fact, the model obtained had an R^2^ value of 0.925, showing a slight worsening given by the inclusion of a large number of observations and, thus, a more dense subdivision of the sample. Other regression algorithms were tested to increase the terms of comparison. GBT achieved the best outcome (R^2^= 0.844) among the tested algorithms. However, it still did not outperform the MLR model, which is ultimately the most appropriate model for data processing.

The classification algorithms were evaluated based on their ability to predict the length of hospital stay (LOS) classes. Among these algorithms, RF achieved the highest accuracy of 77%. Furthermore, RF performed particularly well in predicting class 0, which includes women with shorter hospital stays, and class 2, which is all women with prolonged hospital stays, with an F1-score exceeding 0.70. It can be observed that, compared to other machine learning algorithms [53,54,55], RF proved to be a most promising family of classifiers in different classification and regression tasks, generating accurate forecasts and enabling higher model interpretability, especially on a large dataset, exceeding the predictive power of decision trees. However, despite being among the most versatile classifiers and capable of achieving good performances on datasets with different properties and problems at various complexity levels, the literature does not fully agree on the overall superiority of RF [56]. Indeed, in accordance with the literature, in the present study, the RF algorithm was only slightly superior to other methods in the classification task, and it showed the lowest performances in the regression task.

Lastly, NN fitting was used to analyze the dataset. Compared with the value of R obtained from the MLR model, a higher value was obtained with the NN for training and a lower value, albeit slightly, for the validation and test sets. The decrease can easily be justified by dividing the sample into multiple sets, ultimately demonstrating the good performance of the model.

Finally, the application of the *t*-test allowed the highlighting of the independent variables that significantly affect the independent variable. Pre-operative LOS, Cardiovascular disease, Respiratory disorders, Hypertension, Diabetes, Haemorrhage, Multiple births, Obesity, Pre-eclampsia, Complicating previous delivery, Urinary and gynaecological disorders, and Complication during surgery were the variables for which a *p*-value was less than the threshold value of 0.05. The permutation feature importance associated with the best classification algorithm, on the other hand, showed a significant influence only of Pre-operative LOS, while smaller effects were observed for the following predictors: Multiple births, Complicating previous delivery, and Complication during surgery. Apart from Pre-operative LOS, whose link with LOS is easily explained, the effect of other variables has also already been highlighted in the literature. Cegolon et al. [57], for example, in their study also conducted on the Italian territory, show through the implementation of regression models the effect that multiple births and previous delivery have on hospital stay by type of CS. Blumenfeld et al. [58], on the other hand, show how women who have perioperative complications register, in addition to various clinical consequences, the need to stay longer in the hospital.

This study, already in its current state, has several strengths. A large number of patients and readily available clinical and demographic variables—being linked to the hospital discharge form—are included, and different analysis tools are tested, adding classification algorithms and neural networks to the classic regression models. This allows us not only to understand which clinical variables impact LOS but also to have predictive tools that can help healthcare management in planning and cost containment operations [59].

However, this study is not without its limitations. In particular, no methodologies were adopted to balance the dataset regarding the presence/absence of the comorbidities included in the study; in addition, the degree of complexity for these variables was not discussed as it does not have access to medical records, VDs were not included, and finally, the study, although supported by other evidence in the literature being monocentric, does not allow generalization of the results obtained.

## 5. Conclusions

This study analyzed the data of 12360 women who underwent CS at “San Giovanni di Dio e Ruggi d’Aragona” University Hospital of Salerno (Italy). A comprehensive set of independent variables was created to provide a more detailed description of the patients’ clinical conditions, which were used to examine the total LOS. MLR model, four different regression algorithms, five different classification algorithms, and a neural network were tested.

An application so interesting to healthcare management lends itself to several future developments. First, VD could also be analyzed, observing a greater number of years and variables even through the combined analysis of multiple health facilities similar in the territory and population area.

## Figures and Tables

**Table 1:** Hyperparameter selection for each artificial intelligence model.

Algorithms	Hyperparameters
SVM	‘kernel’:(‘linear’, ‘rbf’), ‘C’:[1, 10, 100], cv = 10
RF	‘n_estimators’: [5, 10, 15, 20], ‘max_depth’: [2, 5, 7, 9], cv = 10
DT	‘max_depth’:range(3,20), cv = 10
MLP	‘hidden_layer_sizes’: [(50,50,50), (50,100,50), (100,)], ‘activation’: [‘tanh’, ‘relu’], ‘solver’: [‘sgd’, ‘adam’], ‘alpha’: [0.0001, 0.05],’ learning_rate’: [‘constant’,’adaptive’], cv = 10
NB	‘var_smoothing’: np.logspace(0,−9, num = 100), cv = 10
VC	‘voting technique’: (‘hard’, ‘soft’)

**Table 2:** Tolerance and Variance Inflation Factor for the multiple linear regression model.

Independent Variable	Tolerance	VIF
Age	0.966	1.035
Pre-operative LOS	0.927	1.079
Thyroid disorder	0.988	1.012
Cardiovascular disease	0.969	1.032
Abnormal foetus	0.977	1.023
Respiratory disease	0.990	1.010
Hypertension	0.952	1.051
Diabetes	0.922	1.084
Haemorrhage	0.968	1.034
Brain and retinal disorders	0.989	1.011
Multiple births	0.926	1.080
Obesity	0.955	1.047
Amniotic fluid disorders	0.938	1.067
Stillborn	0.984	1.016
Pre-eclampsia	0.924	1.082
Tumour	0.993	1.007
Complicating previous delivery	0.876	1.141
Urinary and gynaecological disorders	0.988	1.013
Complication during surgery	0.774	1.291

**Table 3:** Effectiveness performance of the multiple linear regression model.

	R	R^2^	R^2^ Adjusted	Std. Error of the Estimate
MLR Model	0.936	0.876	0.876	1.618

**Table 4:** Coefficients and results of *t*-test for the multiple linear regression model.

Variable	Unstandardized Coefficients		Standardized Coefficients Beta	*t*	*p*-Value
	B	Std. Error			
(Constant)	3.352	0.085	-	39.655	0.000
Age	0.006	0.003	0.008	2.383	0.017
Pre-operative LOS	0.989	0.004	0.912	277.175	**0.000**
Thyroid disorder	0.149	0.284	0.002	0.524	0.600
Cardiovascular disease	0.841	0.160	0.017	5.245	**0.000**
Abnormal foetus	−0.090	0.073	−0.004	−1.236	0.217
Respiratory disease	3.643	0.383	0.030	9.503	**0.000**
Hypertension	0.397	0.092	0.014	4.321	**0.000**
Diabetes	−0.383	0.138	−0.009	−2.766	**0.006**
Haemorrhage	1.222	0.164	0.024	7.475	**0.000**
Brain and retinal disorders	0.030	0.187	0.001	0.162	0.872
Multiple births	0.368	0.083	0.015	4.412	**0.000**
Obesity	0.826	0.147	0.018	5.617	**0.000**
Amniotic fluid disorders	0.008	0.049	0.001	0.168	0.867
Stillborn	−0.238	0.194	−0.004	−1.225	0.221
Pre-eclampsia	1.165	0.114	0.034	10.247	**0.000**
Tumour	0.326	0.214	0.005	1.524	0.127
Complicating previous delivery	−0.110	0.033	−0.011	−3.302	**0.001**
Urinary and gynaecological disorders	0.481	0.100	0.015	4.790	**0.000**
Complication during surgery	0.535	0.047	0.041	11.392	**0.000**

**Table 5:** Effectiveness performances of each regression model.

	LR	RF	GBT	XGBoost
R^2^	0.839	0.705	0.844	0.838
Root Mean Squared Error	1.522	2.595	1.495	1.524

**Table 6:** Best parameters.

Algorithms	Accuracy	Best Parameters
RF	0.77	‘max_depth’: 9, ‘n_estimators’: 10
MLP	0.74	‘activation’: ‘tanh’, ‘alpha’: 0.0001, ‘hidden_layer_sizes’: (50, 100, 50), ‘learning_rate’: ‘adaptive’, ‘solver’: ‘adam’
NB	0.74	var_smoothing = 0.004
SVM	0.75	‘C’: 1, ‘kernel’: ‘linear’
DT	0.76	‘max_depth’: 8
VC	0.77	‘voting technique’: hard, ‘weights’: None

**Table 7:** Precision, Recall, and F1-score of the best algorithm.

Algorithms	Class	Precision	Recall	F1-Score
RF	0	0.76	0.97	0.86
	1	0.80	0.25	0.38
	2	0.76	0.70	0.73

**Table 8:** NN Model summary.

	Samples	MSE	R
Training	8652	2.224	0.944
Validation	1854	2.963	0.935
Testing	1854	3.631	0.917

## Data Availability

The datasets generated and/or analyzed during the current study are not publicly available for privacy reasons but are available from the corresponding author upon reasonable request.

## Abbreviations

CS	Caesarean Section
VD	Vaginal Delivery
LOS	Length Of Stay
ML	Machine Learning
MLR	Multiple Linear Regression
DT	Decision Tree
RF	Random Forest
SVM	Support Vector Machine
MLP	Multilayer Perception
NB	Naive Bayes
VC	Voting Classifier
NN	Neural Network
